# Supplementary material for: Modified Epoxy Resin on the Burning Behavior and Mechanical Properties of Aramid Fiber Composite
Source: Materials (Basel). 2024 Aug 13;17(16):4028. doi: 10.3390/ma17164028 (PMC11356078; doi:10.3390/ma17164028)
Supplement: Supplementary file 1 [file materials-17-04028-s001.zip › materials-3111266-supplementary.pdf]

Supporting Materials

Modified epoxy resin on the burning behavior and mechanical properties of aramid fiber composite

Xuke Lan [1, 2], ChenXi Bian [1, 2], Yunxian Yang [1, 2, 3, 4]*, Qi Zhang [1] and Guangyan Huang [1, 2]

[1] Beijing Institute of Technology Chongqing Innovation Center, Chongqing, 401120, P. R. China
[2]National Key Laboratory of Explosion Science and Safety Protection, Beijing Institute of Technology, Beijing 100081, P. R. China
[3] Beijing Institute of Technology Zhuhai, Zhuhai 519088, P. R. China
[4]Advanced Research Institute of Multidisciplinary Sciences, Beijing Institute of Technology, Beijing 100081, P. R. China
* Correspondence: yunxian.yang@bit.edu.cn

Table S1. Results from TGA, DMA and burning tests.

Sample	*$T_{5wt\%}$ (°C)	*T_{max1} (°C)	*T_{max2} (°C)	*Residue (%)	T_g (°C)	LOI (%)	UL-94 (2mm) $\overline{t1+t2}$ / (s)
**EP**	**364**	**377**	-	18.5	-	-	-
AF	530	-	560	39.8	-	-	-
AF/EP	373	376	560	34.0	146	31.5	Burning out
AF/EP/2%EAD	369	376	559	33.6	145	35.0	4+75
AF/EP/5%EAD	359	378	562	39.7	140	37.5	0+36

*T5wt%: temperature at 5wt% mass loss.

*Tmax1 and *Tmax2: temperature at the maximum mass loss of matrix and the maximum mass loss of fiber, respectively.

*Residue: the value at 700 °C.
